# Hidden Melanoma

**DOI:** 10.5826/dpc.1002a47

**Published:** 2020-04-03

**Authors:** Gabriel Salerni, Carlos Alonso

**Affiliations:** 1 Dermatology Department, Hospital Provincial del Centenario de Rosario, Universidad Nacional de Rosario, Argentina; 2 Diagnóstico Médico Oroño, Rosario, Argentina

**Keywords:** melanoma, dermoscopy, melanoma incognito, diagnosis

## Case Presentation

**Figure 1:**
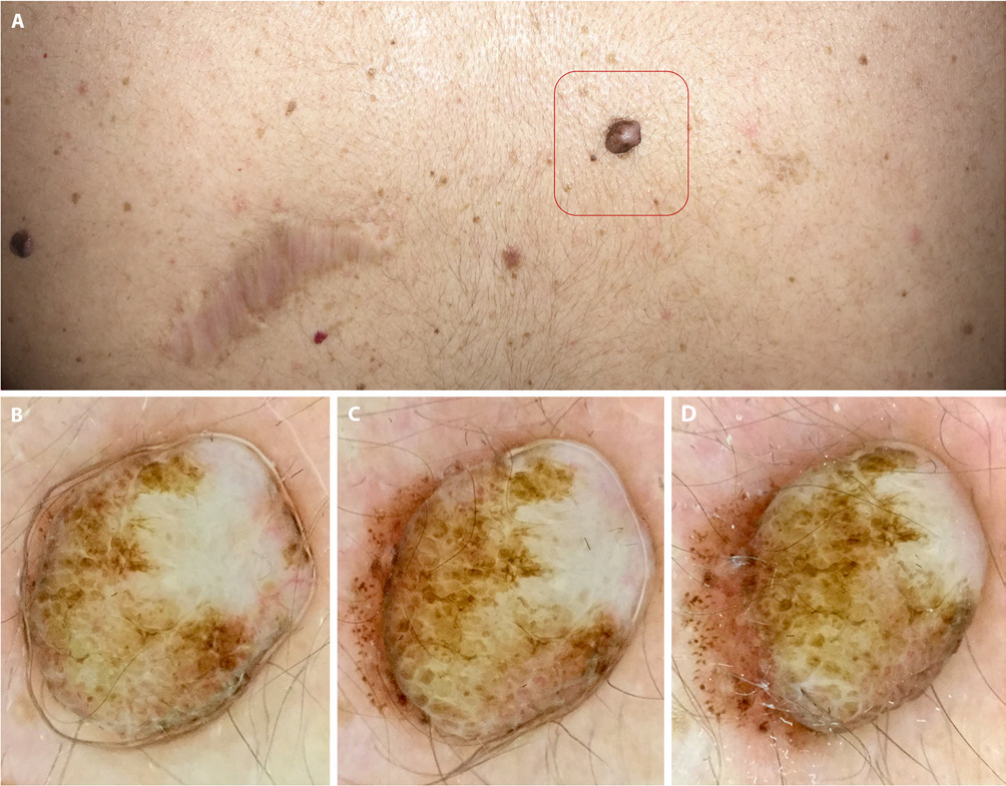
(A) Banal-looking lesion, clinically consistent with dermal nevus, close to melanoma scar. (B–D) With lateral pressure, globules and pseudopods irregularly distributed at the periphery were observed in the base upon dermoscopy.

A 55-year-old woman with a personal history of 2 previous melanomas was referred for assessment. Full-skin examination revealed few nevi, mostly dome-shaped dermal subtype, cherry angiomas, and seborrheic keratosis. On her back, a clinically banal-looking lesion was found close to a melanoma scar (Figure 1A). Dermoscopy initially revealed typical findings pointing to intradermal nevus with cobblestone pattern and area of fibroses attributed to trauma; when lateral pressure was exerted, the basis of the lesion was exposed, revealing pseudopods and globules irregularly distributed at the periphery of the lesion (Figure 1, B–D). The lesion was excised and histopathology reported in situ melanoma associated with dermal nevus.

## Teaching Point

Training and utilization of dermoscopy is recommended for clinicians routinely examining skin lesions. Dermoscopy must be applied to all lesions and not just to those suspicious from a clinical point of view [1]. When facing raised or pedunculated lesions, the base of the lesion must be examined. In this case, dermoscopy additionally provided crucial information for early recognition of a melanoma that might have been overlooked if it had been assessed solely by the naked eye.
